# Angiosarcoma in the femoral bone: A case report of a rare bone tumor

**DOI:** 10.1016/j.ijscr.2024.110124

**Published:** 2024-08-13

**Authors:** Yuni Artha Prabowo Putro, Rahadyan Magetsari, Sharfan Anzhari, Thomas Edison Prasetyo, A. Faiz Huwaidi, Kautsar Akbar

**Affiliations:** a Oncology Divison of Orthopedics and Traumatology Department, RSUP Dr. Sardjito Hospital, Jl. Kesehatan Sendowo No.1, Sleman, 55281, D.I.Yogyakarta, Indonesia; b Orthopedics and Traumatology Department, RSUP Dr. Sardjito Hospital, Jl. Kesehatan Sendowo No.1, Sleman, 55281, D.I.Yogyakarta, Indonesia; c Faculty of Medicine, Public Health and Nursing, Universitas Gadjah Mada, Jl. Farmako, Sendowo, Sekip Utara, Sleman, 55281, D.I.Yogyakarta, Indonesia

**Keywords:** Bone angiosarcoma, Multidisciplinary team approach, Case report, Management, Diagnosis

## Abstract

**Introduction and importance:**

Angiosarcoma is a high-grade endothelial sarcoma rarely found in the bones. Challenges lie in its rareness, high mortality, metastasis, and recurrence. The role of radiotherapy and chemotherapy in managing angiosarcoma remains debatable, while surgery stands out as the optimal treatment for local control. We present a patient with angiosarcoma of the bone, managed through a combination of surgery and radiotherapy.

**Case presentation:**

A 66-year-old male presented with pain on the right distal femur and was unable to weight bearing after lifting a box. The X-ray showed a fracture and lytic lesion. The patient has lost 5 kg of body weight in the last 3 months. The Clinicopathological Conference (CPC) suspects a malignancy process, prompting us for wide resection, biopsy, and ORIF with cement augmentation. Histopathology and immunohistochemistry results confirmed the diagnosis of angiosarcoma. We decide to manage patients with radiotherapy without chemotherapy and regular monthly monitoring. The patient exhibited clinical improvement.

**Discussion:**

Angiosarcoma of the bone tends to have a bad prognosis. Diagnosis might be challenging as it is a rare condition and could be easily confused with another disease. The routine CPC we held was beneficial for the diagnosis and treatment of the patient. The combination of surgery and radiotherapy is yielding positive outcomes.

**Conclusion:**

Angiosarcoma should be considered in patients with osteolytic lesions in the bone. Surgery and radiotherapy are important to local control in patients with angiosarcoma.

## Highlights

• Angiosarcoma sarcoma of the bone poses challenges due to its rareness, high mortality rate, and recurrence.
• Although uncommon, angiosarcoma of the bone should be considered in patients with osteolytic lesions.
• Surgery and radiotherapy have shown promising outcomes in the management of angiosarcoma of the bone.

## 1 Introduction

Angiosarcoma is a rare, high-grade endothelial sarcoma with an incidence of 3/1 million cases and less than 1 % of all sarcomas [[1], [2], [3]]. Though rarely found in the bones, common bone predilection of Angiosarcoma includes the femur and pelvic bone, and rarely found on axial skeleton [2,3]. The cause of angiosarcoma is still unknown, but it is associated with a history of radiotherapy, and bone infarction. Angiosarcoma of the bone can manifest as a unifocal lesion and rarely as a multifocal lesion [2].

Angiosarcoma has a high recurrence rate and metastasis [[4], [5], [6]]. In addition, the small number of cases of angiosarcoma causes a high mortality rate and a low prognosis [3]. Currently, operative therapy has an important role for patients with foAngiosarcoma of the bone [3,7]. However, there is no agreement regarding the need for chemotherapy and radiotherapy for the management of bone angiosarcoma [3,7]. We report a case of angiosarcoma of the bone with the initial manifestation of the pathological fracture of the right femur. This case report was written with SCARE criteria [10].

## 2 Case presentation

A 66-year-old male, a smoker, was referred to our hospital after feeling a sharp pain in his right thigh accompanied by a cracking sound when attempting to lift a container box one day ago. The patient noticed his thigh to be crooked and he was unable to stand properly. There was no injury to other body parts. The X-ray examinations in the local hospital showed a fracture and there were signs of a tumor, so he was referred to our hospital. He had lost 5 kg of body weight during the last 3 months. History of fever was denied and there was no difficulty to ambulate before the incident. Previous history of diagnosed malignancies and radiotherapy were denied nor in his family. The only surgical history was hernia repair operated in 2018. He had mild hypertension that was routinely treated with Amlodipine milligram once a day.

**Fig. 1:**
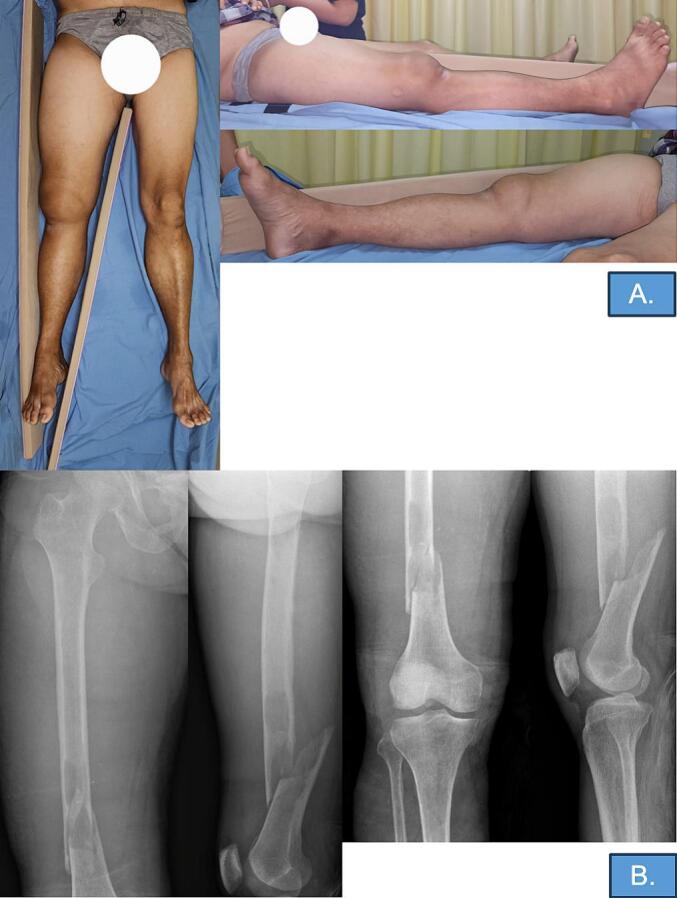
Initial condition of patients A.) Marked swelling can be seen on the right thigh 10 × 12 × 4 cm in size. **B.**) Complete, comminuted fracture of distal third of the right femur with spiral pattern, shortening, proximal fragment was externally rotated while the distal fragment angulated posteriorly, and no bony contact between the fragments.

Physical examination revealed unremarkable vital signs, inability to ambulate, and normal nutritional status. The local examination of the right femur showed marked swelling on the distal femur region 10 × 12 × 4 cm in size with a slightly externally rotated hip. There was no wound, discoloration, shiny appearance, or venous engorgement (Fig. 1). The distal part of the right femur was very tender with a limited range of motion. Laboratory examinations showed anemia (7.2 g/dL), thrombocytopenia (123,000 cells/μL), mildly increased LDH (288 U/L), mild hypocalcemia (2.02 mmol/L), increased Blood Urea Nitrogen (31 mg/dL) and increased creatinine (3.43 mg/dL). X-ray showed a complete, comminuted fracture of the distal third of the right femur with osteolytic lesions around the fracture site (Fig. 1).

**Fig. 2:**
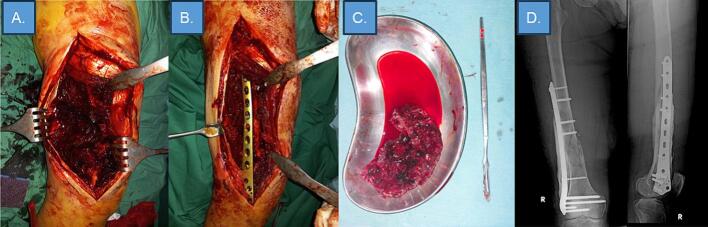
Wide resection, Open biopsy, ORIF and cement augmentation of the right femur.

After the patient was admitted and the anemia was corrected, the patient underwent wide resection, open biopsy, open reduction, and internal fixation (ORIF) with cement augmentation (Fig. 2). The biopsy result was a potential angiosarcoma or rhabdomyosarcoma. The first clinicopathological conference (CPC) for this patient suggested an immunohistochemistry examination with CD31, ERG, and myogenin for confirmation because the histopathology results did not match clinical and radiological findings.

**Fig. 3:**
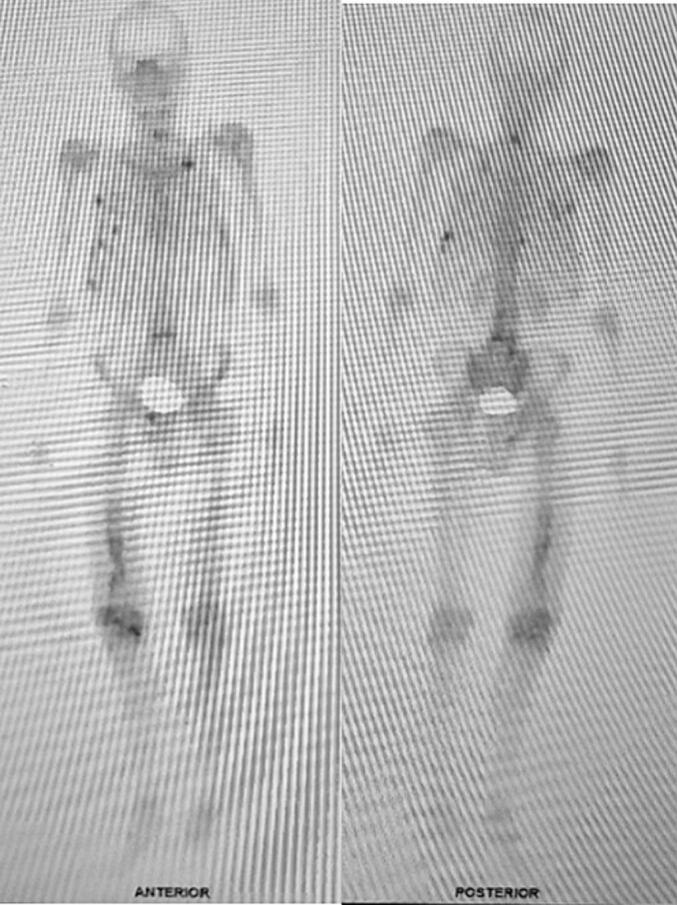
Bone scan examination show multiple hotspot vertebrae and costae, right pubic bone, and right ischium.

**Fig. 4:**
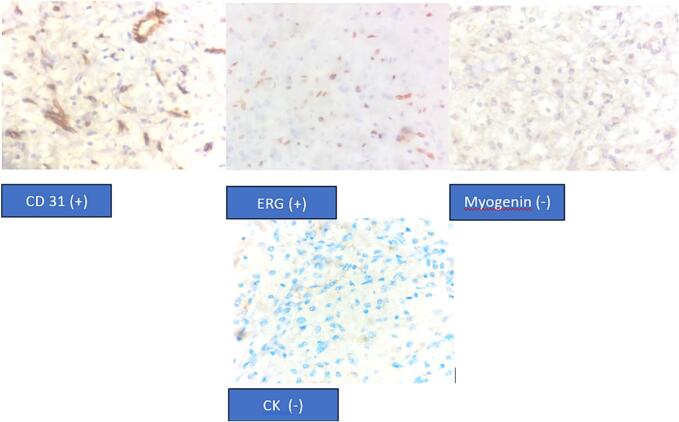
IHC Staining showed that CD31 (+), ERG (+), Myogenin (−) CK (−) and conclude the result suggesting a Angiosarcoma.

Immunohistochemistry showed that myogenin was negative, which may exclude rhabdomyosarcoma, while CD31 and ERG were strongly stained, suggesting Angiosarcoma. In the second CPC for the case, the bone scan result revealed multiple hotspots mainly at vertebrae, costae, right pubic bone, and right ischium (Fig. 3). However, the condition suggested a metastatic bone disease, and then cytokeratin staining and bone scan were further performed. Cytokeratin was negative for all tumor cells (Fig. 4).

The third CPC concluded by diagnosing Angiosarcoma originating from the right femoral bone. We decided to manage the patient with regular monthly monitoring. During the therapy program, the patient reported reduced pain, exhibited clinical improvement, and showed no signs of recurrence. We consistently monitored the patient until 6 months after the surgery.

## 3 Discussion

Currently, the diagnosis and management of angiosarcoma remains challenging. The low incidence rate plus the difficulty of making a diagnosis of Angiosarcoma only from physical and radiological examination is the cause [1,2,4,8]. However, biopsy results that are positive with IHK CD31, ERG, and negative with IHK AE1, CAMTA1, and TFE can confirm the diagnosis of angiosarcoma [7]. This makes a multidisciplinary approach to cancer patients very important, so we regularly hold clinicopathological conferences (CPC) for treating cancer patients*.*

The operative procedures performed on local bone angiosarcoma patients can increase overall survival more than twofold [7]. Meanwhile, in patients with synchronous metastatic angiosarcoma, surgery can be performed with the guarantee achievement of safe margin resection [7]. For chemotherapy, there was no difference in overall survival between patients who underwent chemotherapy or those who did not receive chemotherapy, whether in patients with local or metastatic angiosarcoma of the bone [7]. Radiotherapy combined with surgery can increase overall survival but radiotherapy alone is not effective [4,6]. For cases of multicentric angiosarcoma of the bone, the choice and modality of therapy are still debated [5]. In accordance with the literature, we chose to perform fracture fixation and radiotherapy without chemotherapy. During follow-up, the patient showed clinical improvement, experienced reduced pain intensity, and exhibited no signs of recurrence.

The prognosis for angiosarcoma patients is very low. The 5-year survival rate for angiosarcoma is only 31 % and only 8 % for angiosarcoma of the bone [3,7]. Factors that have been proven to influence the prognosis and survival rate of patients with angiosarcoma of the bone are the stage of the tumor, age, and location of the tumor [7]. Increasing age is associated with increased carcinogenic exposure, changes in biomolecules, a decrease in the immune system, and comorbidities [3].

About 16–44 % of angiosarcoma patients experience metastases [6]. The most common sites for metastasis angiosarcoma are the lungs, liver, and soft tissue. Although rare, hematologic disorders such as persistent thrombocytopenia have been reported in patients with metastatic angiosarcoma to the bone marrow [8,9]. Our patient had persistent anemia and thrombocytopenia, even after local control and surgical removal of the entire tumor in the right femur. The suspicion of bone marrow metastasis cannot be ruled out, because we did not carry out investigations to rule out that possibility.

The radiographic examination will provide a single or regional picture of a well-defined, ill-defined osteolytic lesion with cortical destruction without any periosteal reaction [1]. These findings are not typical for angiosarcoma, but contrast-enhanced CT results can be used for staging and assessing response to therapy in metastatic cases. If surgery is planned, MRI can define soft tissue in detail and infiltration into the vascular area which can determine the type of surgery to be performed [6]. In cases of multiple angiosarcoma, it is recommended to undergo a whole-body imaging examination [5]. We did a bone scan and the results showed multiple hot spot lesions. Although bone scans are very sensitive, they are not specific to define bone lesions as metastatic lesions.

In older patients with multiple bone lytic lesions, the first things that might come to mind are multiple myeloma and metastatic bone disease. However, even though it is rare, angiosarcoma should still be considered as a differential diagnosis. For this reason, a multidisciplinary approach is important in the management of malignant patients. Regarding reports of metastatic or multicentric angiosarcoma, we did not carry out further investigations because no significant difference in prognosis was found [5]. Our limitation lies in the fact that we did not conduct additional investigations to confirm whether our patient had multicentric angiosarcoma of the bone or metastatic bone disease due to angiosarcoma. However, given the rarity of this case, we still present it to contribute valuable information about the angiosarcoma of the bone.

## 4 Conclusion

Even with small cases, angiosarcoma still must be considered as a differential diagnosis in patients with osteolytic lesions in the bone. Surgery and radiotherapy are important to local control in patients with angiosarcoma of the bone but the chemotherapy is still questionable. Because of the poor prognosis of angiosarcoma of the bone, a multidisciplinary approach is important to decide for the patients.

## Consent for publications

Written informed consent was obtained from the patient for publication of this case report and any accompanying images. A copy of the written consent is available for review by the Editor-in-Chief of this journal.

## Ethical approval

This case report doesn't require ethical approval based on the Universitas Gadjah Mada research ethics committee's guidelines. It focuses on a patient's treatment and medical care, not research. Our institution's ethics committee confirmed that this report aligns with routine clinical practice and doesn't involve experimental interventions or additional data collection. We're ready to provide more information if needed, underscoring our commitment to ethical practices.

## Funding

This research received no external funding.

## Author contribution

Y.A. P. P (Conceptualization, Writing- Original Draft, Validation, Investigation), (R.M: Validation, Methodology, Writing- Review & editing, Supervision), (S.A: Writing- Original, Draft, Resources, Validation), (T. E. P: Visualization, Resources, Methodology, Supervision) (A. F. H: Resources, Writing- Original Draft, Visualization, Validation), (K. A: Writing original draft, Methodology, Visualization).

## Guarantor

Y.A.P.P.

## Conflict of interest statement

The authors declare no conflict of interest.

## Data Availability

Supporting data will be available upon reasonable request.
